# Impaired arginine/ornithine metabolism drives severe HFMD by promoting cytokine storm

**DOI:** 10.3389/fimmu.2024.1407035

**Published:** 2024-06-24

**Authors:** Yaozhong Zhang, Qingqing Yang, Qi Peng, Zhihua Tian, Fen Lv, Xiaomei Zeng, Zaixue Jiang, Qingqiu Cheng, Lijun Yang, Baimao Zhong, Xiaomei Lu, Yinghua Zhu

**Affiliations:** ^1^ Department of Genetic Medicine, Dongguan Children’s Hospital Affiliated to Guangdong Medical University, Dongguan, China; ^2^ Department of Medical and Molecular Genetics, Dongguan Institute of Pediatrics, Dongguan, China; ^3^ Department of Genetics, Key Laboratory for Children’s Genetics and Infectious Diseases of Dongguan, Dongguan, China

**Keywords:** severe HFMD, ornithine, cytokine storm, EV71, macrophage

## Abstract

**Introduction:**

The Hand, Foot and Mouth Disease (HFMD), caused by enterovirus 71 infection, is a global public health emergency. Severe HFMD poses a significant threat to the life and well-being of children. Numerous studies have indicated that the occurrence of severe HFMD is associated with cytokine storm. However, the precise molecular mechanism underlying cytokine storm development remains elusive, and there are currently no safe and effective treatments available for severe HFMD in children.

**Methods:**

In this study, we established a mouse model of severe HFMD to investigate the molecular mechanisms driving cytokine storm. We specifically analyzed metabolic disturbances, focusing on arginine/ornithine metabolism, and assessed the potential therapeutic effects of spermine, an ornithine metabolite.

**Results:**

Our results identified disturbances in arginine/ornithine metabolism as a pivotal factor driving cytokine storm onset in severe HFMD cases. Additionally, we discovered that spermine effectively mitigated the inflammatory injury phenotype observed in mice with severe HFMD.

**Discussion:**

In conclusion, our findings provide novel insights into the molecular mechanisms underlying severe HFMD from a metabolic perspective while offering a promising new strategy for its safe and effective treatment.

## Introduction

The hand, foot, and mouth disease (HFMD), primarily caused by enterovirus 71 (EV71), is a prevalent infectious disease among children (1). Since its initial identification in the late 1960s, outbreaks or epidemics of EV71 have been frequently reported worldwide in various countries. Each outbreak is accompanied by a significant number of severe HFMD cases, leading to fatalities among infected children (2–4). Severe HFMD has emerged as a global health threat for infants and young children. Currently, there is no effective and safe therapy available to treat severe HFMD except for glucocorticoids (5). Therefore, it is crucial to uncover the molecular mechanism and explore safe and effective treatment strategies for managing severe HFMD.

The clinical manifestations of severe HFMD include pulmonary edema, acute flaccid paralysis, rapid elevation in blood glucose levels, and limb paralysis (6). Numerous studies have demonstrated that these life-threatening clinical symptoms may be attributed to a cytokine storm triggered by excessive release of inflammatory cytokines and chemokines (7). Pathological examination revealed severe tissue damage and significant infiltration of inflammatory cells at the sites of severe injury (8). Evidence from serum analysis of patients with severe HFMD showed a marked increase in the levels of various cytokines, including TNF-α, IL-6, MCP-1, and CXCL10 (9). Previous studies have confirmed that macrophages are the primary producers of various pro-inflammatory factors such as IL-6, IL-1β, TNF-α, and CXCL10 (10). Furthermore, it has been demonstrated that amino acid metabolism can regulate the secretion of pro-inflammatory cytokines in macrophages (10).

Amino acids, being vital nutrients in the human body, play a crucial role in maintaining immune system homeostasis. Numerous studies have demonstrated that various amino acids and their metabolites, such as branched-chain amino acids, glutamine, methionine, and arginine, exert influence on immune cell activation and inflammatory factor secretion (11, 12).

Although recent studies have demonstrated significant alterations in circulating amino acid levels in the host due to various viral infections, such as SARS-CoV-2 and African swine fever virus infection (13–15), there is currently no study elucidating the changes in amino acid metabolism and its role in the development of cytokine storm during severe HFMD. In this study, our focus lies on investigating the molecular mechanism of severe HFMD through examining macrophage-associated cytokine storm and alterations in amino acid metabolism, with an aim to explore safe and effective treatment strategies for managing severe HFMD.

## Materials and methods

### Ethics statement

In this study, inbred, specific-pathogen-free BALB/c mice were employed as experimental animals. Ethical approval for all animal experiments was obtained from the Life Science Ethics Review Board of Guangdong Medical University (Permission No. GDY2302210).

### Mice

BALB/c mice were procured from the Guangdong Medical Laboratory Animal Center and were housed in a specific-pathogen-free facility at the Affiliated Dongguan Children’s Hospital of Guangdong Medical University. The mice were maintained on a 12-hour light/dark cycle with ad libitum access to food and water.

### Viral infection

Three-day-old BALB/c mice were intraperitoneally inoculated with 1*10^6^ TCID50 of EV71 (strain 888/GZ/CHN/2008, GU190180.1). Control mice received an equal volume of the culture supernatant of human rhabdomyosarcoma (RD) cells. Daily monitoring for mortality and morbidity occurred for two weeks. Disease scores were assigned based on specific criteria: 0, healthy; 1, lethargy, piloerection, and tremor; 2, persistent weight loss and decreased activity; 3, kyphosis and limb weakness; 4, limb paralysis and deformity; 5, dying or death. Survival curves and disease scoring graphs were established using GraphPad Prism. Statistical analysis employed the Log-rank (Mantel-Cox) Test and two-way ANOVA, with a significance threshold set at P<0.05.

### Tissue collection and preparation

Lungs, skeletal muscles, spinal cords and pancreas were collected at 7 days post-infection (dpi) for subsequent analyses. Tissues were subjected to RNA-seq sequencing (n = 3 for control-infected mice; n = 3 for EV71-infected mice), immunohistochemical (IHC) staining (n = 3 for control-infected mice; n = 3 for EV71-infected mice), and Real-time qPCR analysis (n = 3 for control-infected mice; n = 3 for EV71-infected mice). The IHC and Real-time qPCR data represent three separate experiments.

### Immunohistochemical analysis

At 7 dpi, control (n=3) and infected mice (n=3) were euthanized. Lungs, skeletal muscles, and spinal cords were fixed in 10% paraformaldehyde for 48 hours. Paraffin-embedded tissues were sectioned (4μm) and stained with hematoxylin and eosin (H&E). Tissue sections were dewaxed, dehydrated, and antigen-repaired using the pressure cooker method in sodium citrate solution. Sections were then incubated with 3% H_2_O_2_ for 10 minutes, primary antibody for 16 hours, followed by a streptavidin-based detection system. Sections were observed under a light microscope after counterstaining. Control sections were incubated with PBS instead of antibody to check for the absence of non-specific effects due to the secondary antibody (biotinylated anti-Rabbit IgG). The primary antibody used in this study were anti-F4/80 monoclonal antibody (1:200 dilution, Cell Signaling Technology), anti-EV71 monoclonal antibody (1:400 dilution, SIGMA), anti-ODC1 monoclonal antibody (1:200 dilution, Abcam).

### Cell culture

THP-1 cells (Human myeloid leukemia mononuclear cells, ATCC number: TIB-202) were cultured in RPMI-1640 medium supplemented with 10% fetal bovine serum at 37°C with 5% CO2 in a humidified incubator.

### RNA interference

5*10^5^ THP cells were inoculated on a 6-well plate, cultured with RPMI-1640 medium containing 10%FBS and 150nmol/ml PMA for 24 h, and then transfected with ODC1siRNA(A10001, GenePharma) according to according to the manufacturer’s siRNA operating instructions.

### Peripheral blood mononuclear cell isolation

Mouse peripheral blood was obtained by blood sampling through the eye frame, and mouse peripheral blood mononuclear cells were obtained using the mouse peripheral blood mononuclear cell extraction kit(TBD2011M) according to the manufacturer’s instructions.

### Western blot analysis

Protein samples were extracted from tissues using RIPA buffer supplemented with protease and phosphatase inhibitors. Protein concentrations were determined using a BCA protein assay kit. Equal amounts of protein were separated by SDS-PAGE and transferred to PVDF membranes. Membranes were blocked with 5% non-fat milk and probed with primary antibodies overnight at 4°C. After washing, membranes were incubated with HRP-conjugated secondary antibodies. Protein bands were visualized using an enhanced chemiluminescence (ECL) detection system. The primary antibody used in this study was anti-ODC1 monoclonal antibody (1:1000 dilution, Abcam).

### ELISA

Levels of specific proteins in tissue homogenates were determined using commercially available ELISA kits following the manufacturer’s instructions. Absorbance was measured at the appropriate wavelength using a microplate reader. Standard curves were generated to quantify the concentration of target proteins.

### RNA extraction, reverse transcription, and qPCR analysis

Total RNA was extracted using EZ-press RNA Purification Kit (EZBioscience) and reverse transcribed using Color Reverse Transcription Kit for RT-PCR (EZBioscience). Quantitative real time-PCR (qPCR) was performed using 2X SYBR Green qPCR Master Mix (EZBioscience) on Roche LightCycler480. GAPDH was used for normalization of mRNA. All reactions were carried out in triplicate and analysis was carried out using 2-ΔΔ Ct method. The primer sequences are shown in **Supplementary Table 1**.

## Results

### Establishment of severe HFMD mouse model with EV71 infection

**Figure 1:**
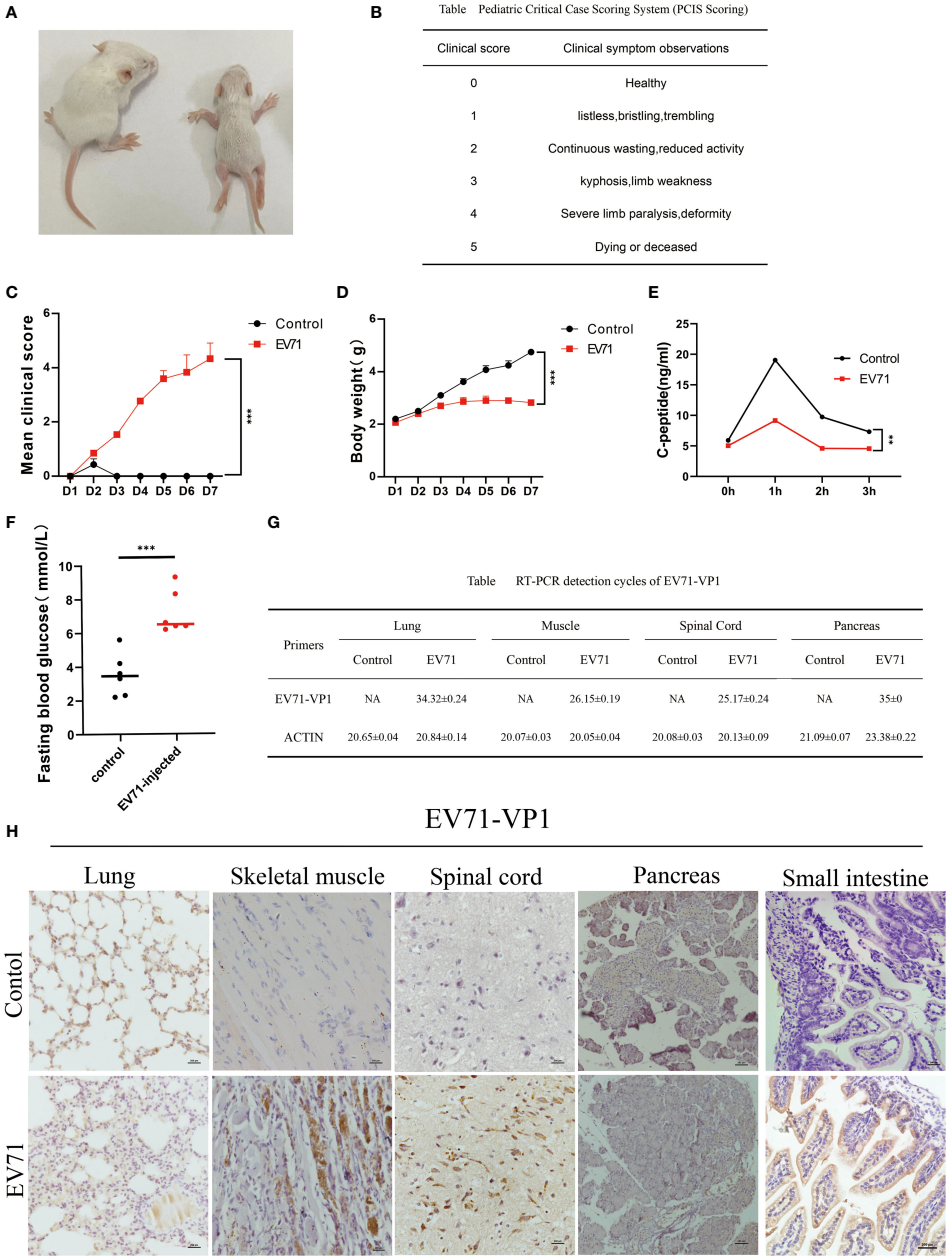
Severe HFMD mouse model established by EV71 virus infection. **(A)** Picture of mice on the third day of abdominal injection of EV71 virus(Left is the control group, right is the EV71 infection group; **(B)** Standard table for pediatric critical case scoring method(PCIS); **(C)** Score the mice according to the PCIS scoring criteria. The line chart shows the changes in PCIS scores of mice after injection of EV71virus; **(D)** The weight of mice injected with EV71 virus; **(E)** The C-peptide value in serum detected using ELISA technology; **(F)** Fasting blood glucose on the third day of injection of EV71 virus solution; **(G)** qPCR detecting the expression of EV71-VP1 in lung tissue, muscle tissue, spinal cord tissue, and pancreatic tissue; **(H)** IHC detecting the expression of EV71-VP1 in lung, muscle, spinal cord, pancreatic and small intestine. (*p<0.05, **p<0.01, ***p<0.001).

In this project, we established a mouse model by infecting 3-day-old mice with 1*10^6^ TCID50 of EV71 strain (888/GZ/CHN/2008) via the IP route. The infected mice were subjected to daily weight measurements and clinical symptom observation for seven days (**Figure 1A**). The health status was meticulously recorded based on clinical symptom scores (**Figure 1B**). Our study revealed a progressive onset of clinical manifestations in EV71 infected mice, characterized by gradual weight loss and muscle weakness emerging on the second day post-infection, with paralysis becoming evident by the third day (**Figures 1C, D**). C-peptide and fasting blood glucose levels were quantified in infected mice, demonstrating a marked reduction in C-peptide concentrations and a conspicuous elevation in fasting blood glucose levels (**Figures 1E, F**). Viral loads were assessed in various tissues including lungs, small intestine, muscles, spinal cord and pancreas. The results showed that EV71 was strongly positive only in severely injured spinal cord and muscle tissues but not other severely injured tissues such as lung or pancreas in severe HFMD mice (**Figure 1G**). Consistent with this, Immunohistochemical(IHC) results showed strong positive EV71 VP1 protein in the spinal cord and muscle tissue of EV71 infected mice (**Figure 1H**). In addition, our results also showed that EV71 VP1 protein was strongly stained in the intestinal lumen of EV71-infected mice, which confirmed the property of EV71 transmission through the digestive tract (**Figure 1H**).

### Cytokine storm is associated with tissue damage in severe HFMD mice

**Figure 2:**
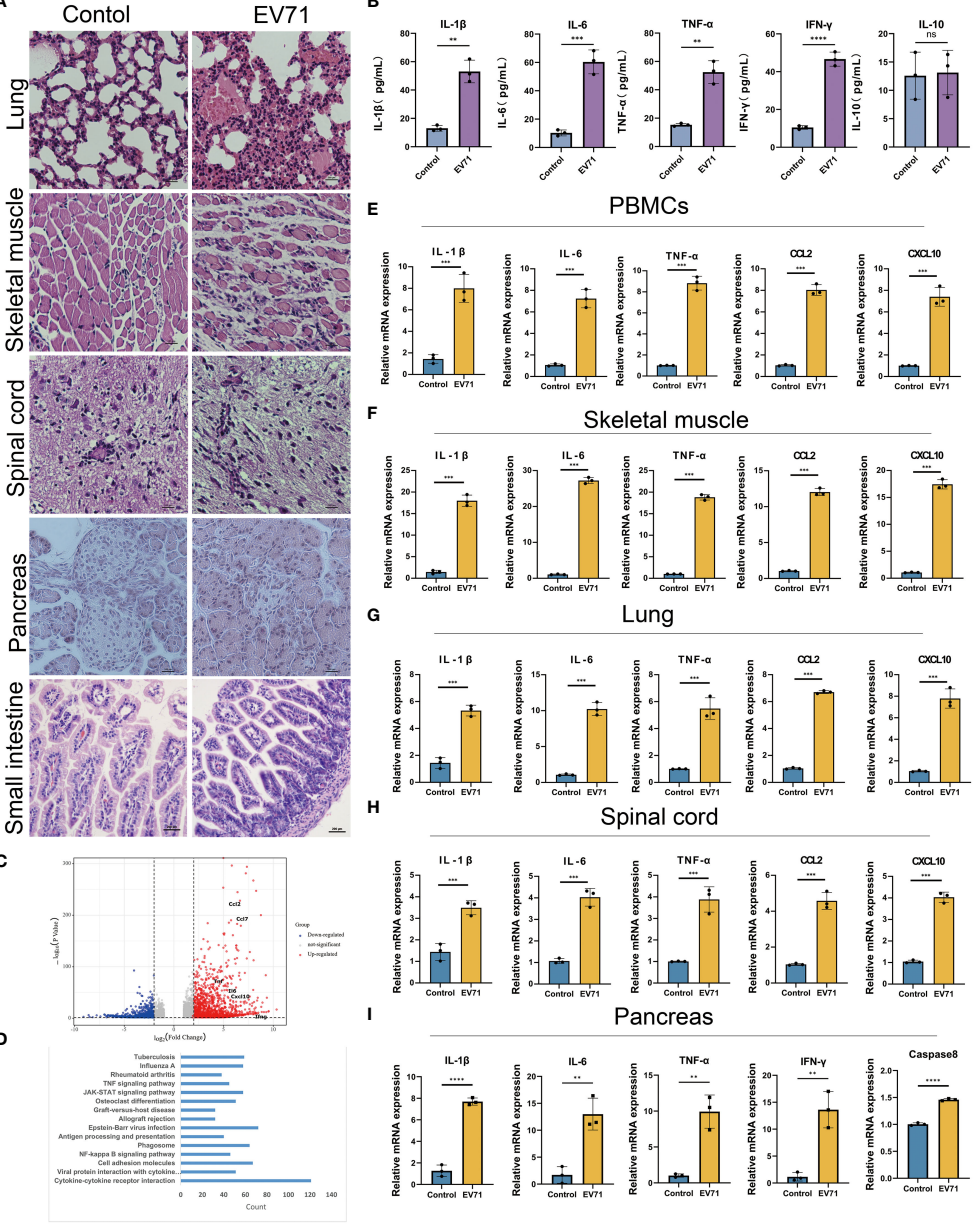
EV71 infection causes inflammatory cytokine storm. **(A)** Histopathological examination (HE) assessing tissue(lung, muscle, spinal cord, pancreatic and small intestine) damage in the severe HFMD mice; **(B)**The expression of relevant inflammatory cytokines in the serum of severe HFMD mice detected by ELISA; **(C)** Perform genetic difference analysis on mice with severe HFMD. The volcano map shows the distribution of differentially expressed genes; **(D)** Kegg enrichment analysis showed pathway enrichment; **(E–I)** qPCR examining the expression of relevant inflammatory factors in tissues of severe HFMD mice. (**p<0.01, ***p<0.001, ****p<0.0001, ns = not significant).

The previous studies have demonstrated that severe HFMD is frequently accompanied by extensive tissue damage and aberrant secretion of inflammatory factors. Hence, we initially assessed the tissue damage status and serum levels of inflammatory factors in mice with severe HFMD using Hematoxylin and Eosin (HE) staining and Elisa experiment. We observed an extensive tissue damage in the lungs, skeletal muscles, and spinal cord of the severe HFMD mice (**Figure 2A**), and a significant increase of serum levels of cytokines including IL1-β, IL-6, TNF-α, IFN-γ and IL-10 (**Figure 2B**). To elucidate the molecular mechanism of tissue damage in severe HFMD mice, we conducted transcriptome sequencing using the injured muscle tissue, and the sequencing results showed a significant upregulation of key cytokines (IL-1β, TNF-α, IL-6, CXCL10, CCL2) in the severe group (**Figure 2C**). Pathway enrichment for the up-regulated genes showed that multiple cytokine-related pathways were significantly enriched (**Figure 2D**). Furthermore, qPCR analysis revealed markedly elevated expression levels of inflammatory factors, including IL-6, TNF-α, IL-1β, CCL2 and CXCL10, in the PBMCs, lungs, skeletal muscles, spinal cord and pancreas tissues of the severe HFMD mice compared to the control group (**Figures 2E–I**).

It is well known that excessive release of inflammatory factors can cause tissue damage. In this study, our results suggest that cytokine storm caused by abnormal secretion of multiple cytokines may contribute to the development of severe clinical manifestations caused by EV71 infection.

### Macrophages contribute to the formation of cytokine storm caused by EV71 infection

**Figure 3:**
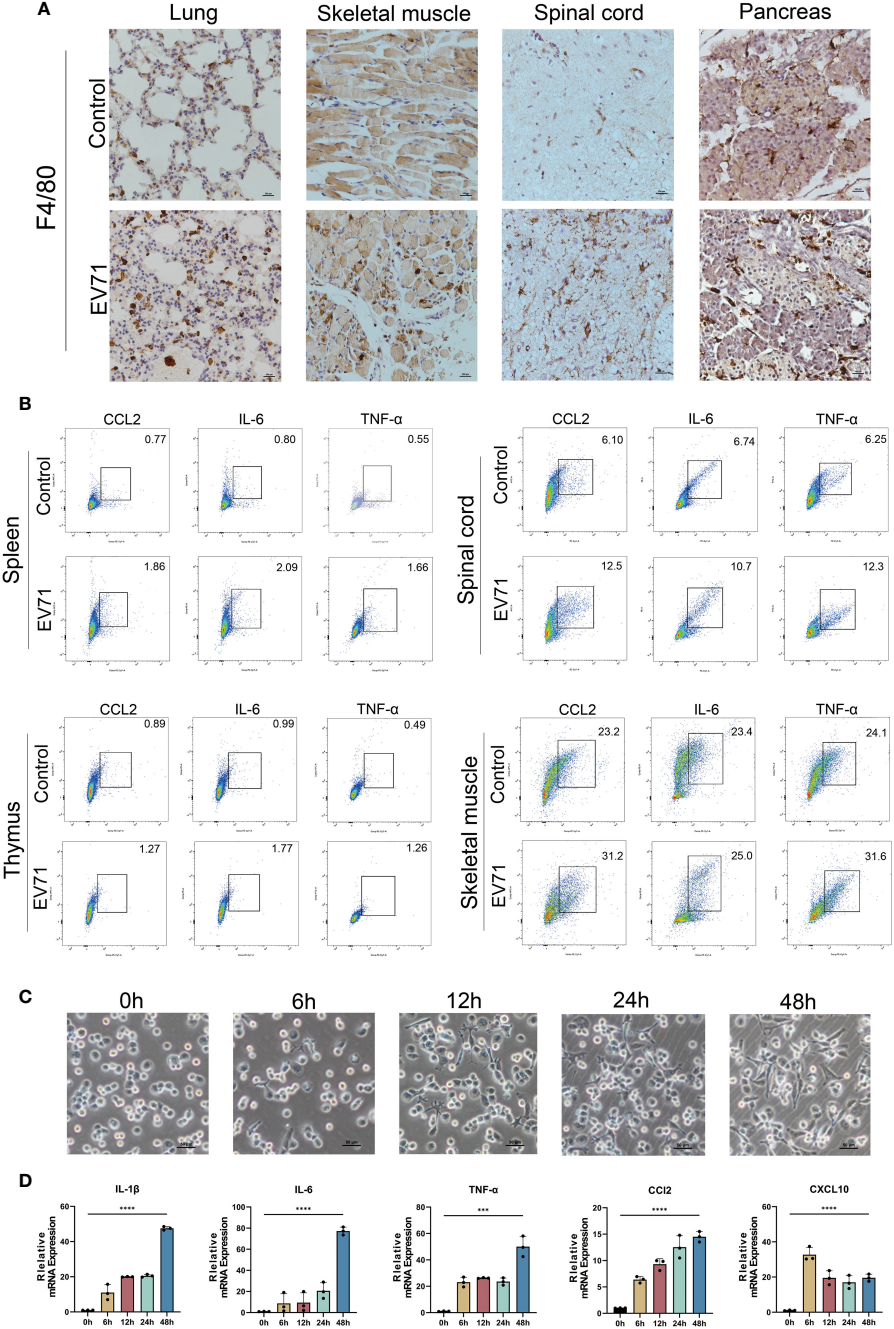
Cytokine storm is associated with extensive macrophage infiltration. **(A)** Immunohistochemistry (IHC) assessing the infiltration of macrophages using the macrophage marker F4/80 in the damaged tissues of severe HFMD mice; **(B)** Flow cytometry examining the expression of inflammatory cytokines in macrophages of severe HFMD mice; **(C)**Morphological changes in THP1 cells infected with EV71(0h,6h,12h,24h,48h); **(D)** qPCR examining the expression of inflammatory cytokines in the EV71-infected macrophage model. (***p<0.001, ****p<0.0001).

Since macrophages are major producers of inflammatory cytokines, we first investigated the infiltration of macrophages in the damaged tissues of severe HFMD mice using the macrophage marker F4/80. The findings revealed a significantly higher density of infiltrating macrophages in the lung, skeletal muscle, spinal cord, and pancreas of severe HFMD mice compared to the control group (**Figure 3A**). To confirm the correlation between macrophages and inflammatory cytokine storms, we examined the expression of inflammatory cytokines in macrophages of severe HFMD mice by flow cytometry. The results of flow cytometry showed that the levels of CCL2, IL-6 and TNF-αin macrophages in spinal cord, muscle, spleen and thymus of severely HFMD mice were significantly increased. The levels of macrophage-derived inflammatory factors such as CCL2, IL-6 and TNF-α in the spinal cord, muscle, spleen and thymus of severe HFMD mice were significantly increased (**Figure 3B**).

To further validate the predominant role of macrophages as the primary source of excessive inflammatory factors in severe HFMD, we established an *in vitro* model of EV71 infection using THP1 cells and assessed alterations in the expression profile of inflammatory factors post-infection. Our experimental findings demonstrated that EV71 infection not only induced significant morphological changes in macrophages but also stimulated robust secretion of various proinflammatory cytokines, including IL-6, TNF-α, IL-1β, CCL2, and CXCL10 (**Figures 3C, D**).

### EV71 infection resulted in impaired arginine/ornithine metabolism

**Figure 4:**
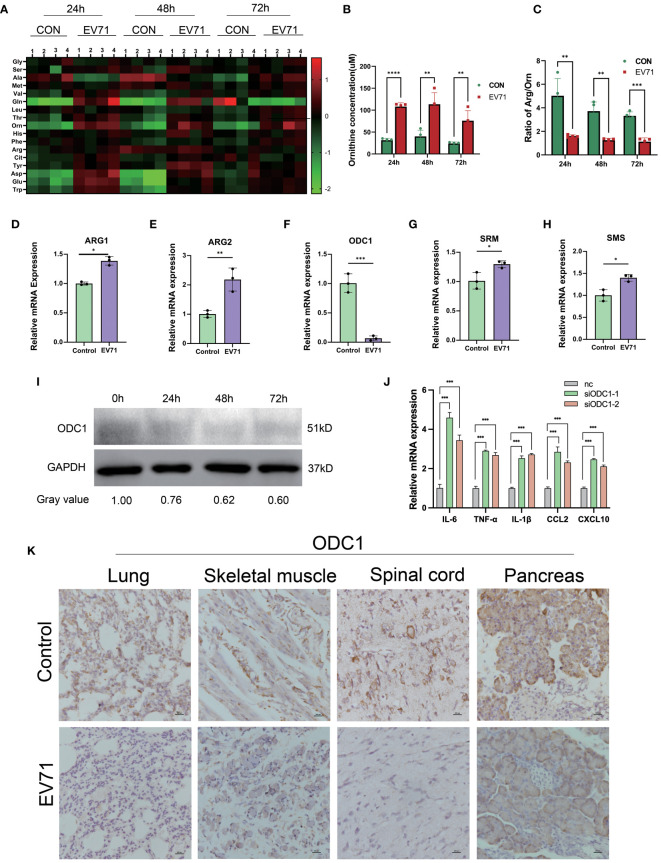
Altered arginine metabolism induced by EV71 infection. **(A)** Heat map showed the changes of amino acids profile in EV71-infected mice; **(B)** Changes of ornithine concentration in peripheral blood of EV71-infected mice; **(C)** Changes of ornithine/arginine ratio in peripheral blood of EV71-infected mice; **(D–H)** qPCR assay detecting the expression of genes involved in arginine metabolic pathways. **(I)** Western blot examining the expression of ODC1 in macrophages treated with EV71; **(J)** qPCR examining the expression of cytokines in macrophages treated with ODC1 siRNA; **(K)** IHC examining the expression of ODC1 in severely damaged mouse tissues. (*p<0.05, **p<0.01, ***p<0.001, ****p<0.0001).

Numerous studies have shown that amino acid metabolism reprogramming can participate in the regulation of macrophage inflammatory factor secretion. In order to reveal the molecular mechanism of severe HFMD from the perspective of amino acid metabolism, we used mass spectrometry to analyze the changes of serum amino acid profiles at different time points after EV71 infection. We observed a sustained increase in serum ornithine levels within 72 hours after EV71 infection as compared to the control group mice (**Figures 4A–C**). In order to investigate the underlying factors contributing to the elevation of ornithine levels induced by EV71 infection, we conducted a qPCR experiment to assess alterations in the expression of genes associated with the arginine/ornithine metabolic pathway, including ARG1, ARG2, ODC1, SRM, SMS. Our findings revealed a significant decrease in ODC1 expression (**Figures 4D–H**), and ODC1 was reported the rate-limiting factor in polyamine synthesis. Additionally, we examined changes in ODC1 protein levels following 24h, 48h and 72h of EV71 infection in THP1 cells and observed a continuous decline within 72 hours (**Figure 4I**). qPCR analysis of macrophages treated with ODC1 siRNA revealed an upregulation in the expression of inflammatory cytokines, including IL-6, TNF-α, IL-1β, CXCL10, and CCL2 (**Figure 4J**). Consistently with these results, immunohistochemical investigations demonstrated a notable reduction in ODC1 expression within damaged lung tissue as well as skeletal muscle, ganglion and pancreas samples obtained from EV71-infected mice (**Figure 4K**). These results suggest that the blockage of ornithine metabolism caused by down-regulation of ODC1 is related to the severe clinical HFMD caused by EV71 infection.

### Therapeutic effects of ornithine metabolites in severe HFMD mice

Numerous studies have demonstrated that polyamines inhibit the expression of inflammatory factors. Therefore, the significant reduction in ODC1 expression leading to polyamine deficiency may be the primary cause of cytokine storm induced by EV71 infection, which suggests that inhibiting cytokine storm caused by EV71 infection through polyamine is a potential therapeutic strategy for severe HFMD.

**Figure 5:**
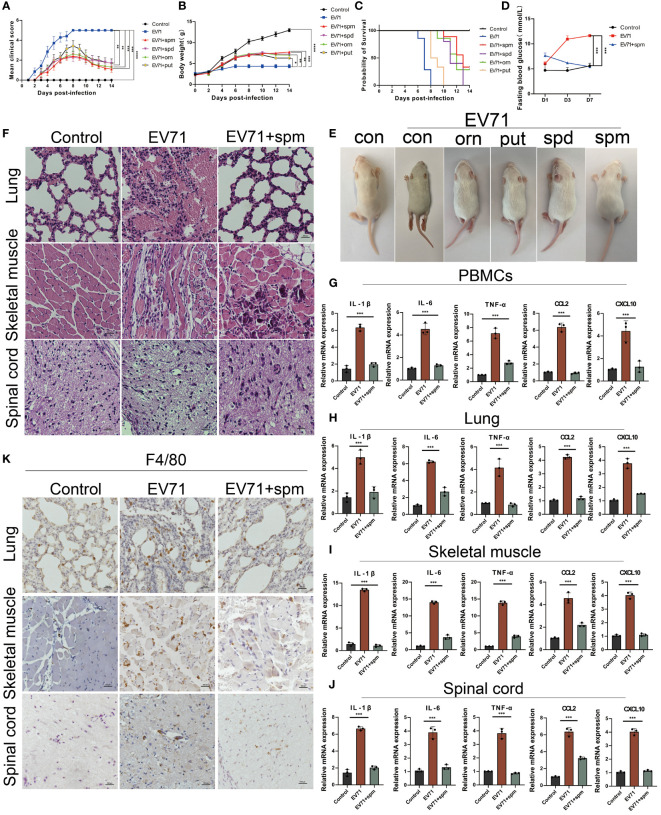
The arginine metabolite spermine can effectively treat severe HFMD in mice. **(A)** PCIS clinical scoring for evaluating the efficacy of amino acid metabolite therapy in mice(ornithine:orn; putrescine:put; spermidine:spd; spermine: spm); **(B)** Weight of mice treated with arginine metabolites; **(C)**Survival analysis evaluated mice treated with arginine metabolism therapy; **(D)** Fasting blood glucose levels in mice treated with spermine; **(E)**Images of HFMD mice treated with different arginine metabolites; **(F)** HE staining assessing tissue damage in severe HFMD mice treated with spermine; **(G–J)** qPCR assay examining the expression of inflammatory cytokines in PBMCs, Spinal Cord, Lungs, and Muscles; **(K)** IHC assessing the infiltration of macrophages using the macrophage marker F4/80 in the tissues of severe HFMD mice. (*p<0.05, **p<0.01, ***p<0.001, ****p<0.0001).

In this study, we investigated the therapeutic effects of polyamines on severe HFMD in both EV71-infected THP1 cell model and mouse model. After treatment of EV71-infected THP1 cells with ornithine metabolites, we found that polyamines, especially spermine, not only changed macrophage morphology, but also significantly inhibited the expression of multiple proinflammatory cytokines including IL-1β, IL-6, TNF-α, IFN-γ, CCL2 and CXCL10 (**Supplementary Figures 1A, B**). Consistent with this, the administration of polyamines, particularly spermine, significantly improved the paralysis symptoms in the hind limbs of severe HFMD mice, and the results from body weight, clinical scores, blood glucose levels, and prognostic survival analyses collectively demonstrate that spermine significantly improved the severe phenotype observed in EV71-infected mice (**Figures 5A–E**). Furthermore, HE staining and immunohistochemistry results showed that spermine treatment significantly reduced tissue damage and macrophage infiltration in lung, skeletal muscle and spinal cord of severe HFMD mice (**Figures 5F, K**). Additionally, the results of cytokine detection showed that spermine could significantly reduce the expression of multiple inflammatory factors in injured tissues and peripheral blood monocytes (**Figures 5G–J**).The findings suggest that spermine holds promise as a safe and efficacious novel therapeutic approach for managing severe HFMD.

## Discussion

In this study, our findings demonstrate for the first time that disruption of ornithine metabolism, leading to suppression of polyamine synthesis, plays a pivotal role in driving cytokine storm in severe HFMD. Furthermore, considering the inhibitory effects of polyamines on multiple inflammatory factors, we evaluated the therapeutic potential of polyamines in severe HFMD mice and observed significant reversal of the severe phenotype induced by EV71 infection with polyamines, particularly spermine. This discovery may provide a more safe and effective therapeutic strategy for individuals with severe HFMD in the future.

In order to elucidate the molecular mechanism underlying severe HFMD, we established a mouse model of EV71 infection and observed strong positive expression of the EV71 capsid protein VP1 in severely damaged spinal cord and muscle tissues, while no such expression was detected in other severely affected tissues such as lung and pancreas in severe HFMD mice. This observation was accompanied by elevated levels of inflammatory cytokines and glucose in serum, which closely resembled those reported in previous cases of human severe HFMD (16, 17). Both our findings and previous studies suggest that a cytokine storm resulting from excessive secretion of inflammatory factors may play a crucial role in multi-organ damage among individuals with severe HFMD (18). Despite extensive research on severe HFMD caused by EV71 infection, to the best of our knowledge, this study is the first to report pancreatic tissue damage induced by EV71 infection, providing a plausible explanation for the increased blood glucose levels observed in patients with severe HFMD.

It is widely recognized that amino acid metabolism plays a crucial role in the regulation of both innate and adaptive immunity. In recent years, various viral infections, such as SARS-CoV-2 (19) and influenza virus (20), have been shown to induce alterations in host cell amino acid metabolism. Cheng et al. demonstrated significant metabolic changes, particularly in glutathione, glutamate, and aspartate levels, in EV71-infected Vero cells which were dose-dependent (21). However, this study was conducted solely using an *in vitro* cell model of EV71 infection without validation *in vivo* models, thus limiting its ability to accurately reflect metabolic changes within the host. In our current study, we analyzed the amino acid metabolic profiles at different time points in severe HFMD mice. Consistent with Cheng’s findings, we observed significant increases in serum glutamate and aspartate levels at 24h and 48h post-EV71 infection. Interestingly, we also noted a continuous elevation of serum ornithine levels up to 72h after EV71 infection with the most pronounced increase.

In mammals, Ornithine is an important intermediate in the arginine/polyamine metabolic pathway. Arginine undergoes enzymatic hydrolysis by arginase1 (ARG1) and arginase2(ARG2) to yield ornithine (22, 23). Ornithine is in turn converted to putrescine(PUT), spermidine (SPD) and spermine (SPM) under the action of ornithine decarboxylase1(ODC1), spermidine synthase (SRM) and spermine synthase (SMS) (24). It has been well established that ODC1 serves as the rate-limiting enzyme for polyamine synthesis. To investigate the molecular mechanism underlying elevated levels of ornithine caused by EV71 infection, we examined gene expression related to arginine/ornithine/polyamine metabolism in EV71-infected macrophages. Our findings revealed upregulation of ARG1 and ARG2 while ODC1 was significantly downregulated. Additionally, we observed decreased expression of ODC1 in injured tissues from severe HFMD mice. Therefore, our results provide preliminary insight into elevated levels of ornithine found in severe HFMD mice and suggest a potential polyamine deficiency due to reduced ODC1 expression.

In recent years, the significant roles of SPD and SPM in cancer proliferation, tissue injury protection, viral infection, and anti-inflammation have been extensively demonstrated (25–27). Spermine has been reported to exert antiviral effects by stabilizing DNA binding with the DNA sensor cGAS (28). Previous studies have shown that polyamines, particularly spermine, inhibit the synthesis of pro-inflammatory cytokines such as IL-6, CXCL10, IL1-β and TNF-α through suppression of TLRs/STAT1 and NFkB signal pathway activation or promotion of STAT6/PPARγ signal pathway activation (29, 30). Huang and Li et al. reported elevated serum levels of SPD and SPM in EV71-infected children in 2023 (31). However, their study focused on mild cases where patients recovered well. The increase in serum SPM levels may contribute to patient recovery by inhibiting excessive secretion of inflammatory factors. Our findings suggest that a cytokine storm resulting from insufficient spermine secretion may play a crucial role in severe HFMD development. Therefore, we further investigated whether polyamines could rescue the severe phenotype of HFMD mice. Our results indicated that polyamines, particularly spermine (SPM), significantly suppressed the expression of pro-inflammatory factors and improved paralysis symptoms in severe HFMD mice, which is the first time to reveal the therapeutic effect of spermine against severe HFMD.

Although this study has initially confirmed the therapeutic effect of spermine on severe HFMD, there are still some shortcomings in this study, such as the molecular mechanism of spermine inhibiting inflammatory factor storm has not been revealed. Recent studies have shown that TRIM29-PERK signaling pathway plays an important role in promoting CBV-induced myocarditis. Inhibition of TRIM29-PERK signaling pathway can reverse the myocarditis caused by inflammatory cytokines such as IL-6, IL1-β and TNF-α (32). This suggests that the suppression of TRIM29-PERK signaling pathway may be one of the molecular mechanisms by which spermine inhibits the inflammatory cytokine storm induced by EV71 infection, which will be further explored in our future studies.

## Data availability statement

The raw data supporting the conclusions of this article will be made available by the authors, without undue reservation.

## Ethics statement

Ethical approval was not required for the studies on humans in accordance with the local legislation and institutional requirements because only commercially available established cell lines were used. The animal study was approved by The Life Science Ethics Review Board of Guangdong Medical University. The study was conducted in accordance with the local legislation and institutional requirements.

## Author contributions

YZZ: Writing – original draft, Data curation, Investigation, Methodology, Project administration, Validation. QY: Writing – original draft, Data curation, Formal analysis, Investigation, Software, Validation. QP: Writing – original draft, Investigation, Project administration. ZT: Writing – original draft, Investigation. FL: Writing – original draft, Project administration. XZ: Writing – original draft, Project administration. ZJ: Writing – original draft, Project administration. QC: Writing – original draft, Project administration. LY: Writing – original draft, Investigation. BZ: Writing – review & editing, Funding acquisition, Resources, Supervision. XL: Writing – review & editing, Supervision. YHZ: Writing – review & editing, Funding acquisition, Investigation, Project administration, Supervision.

## Supplementary material

Supplementary Figure 1 Arginine metabolites inhibit cytokine storms in macrophages. **(A)**Arginine metabolites(ornithine:orn; putrescine:put; spermidine:spd; spermine:spm) affect the microscopic morphology of macrophages treated with EV71; **(B)**Arginine metabolites reduced the expression of EV71-induced IL-1β, IL-6, TNF-α, IFN-γ, CCL2 and CXCL10 in macrophages. (*p<0.05,**p<0.01,***p<0.001, ****p <0.0001).
