# Supplementary figures and images for: Impaired arginine/ornithine metabolism drives severe HFMD by promoting cytokine storm
Source: Front Immunol. 2024 Jun 24;15:1407035. doi: 10.3389/fimmu.2024.1407035 (PMC11228176; doi:10.3389/fimmu.2024.1407035)

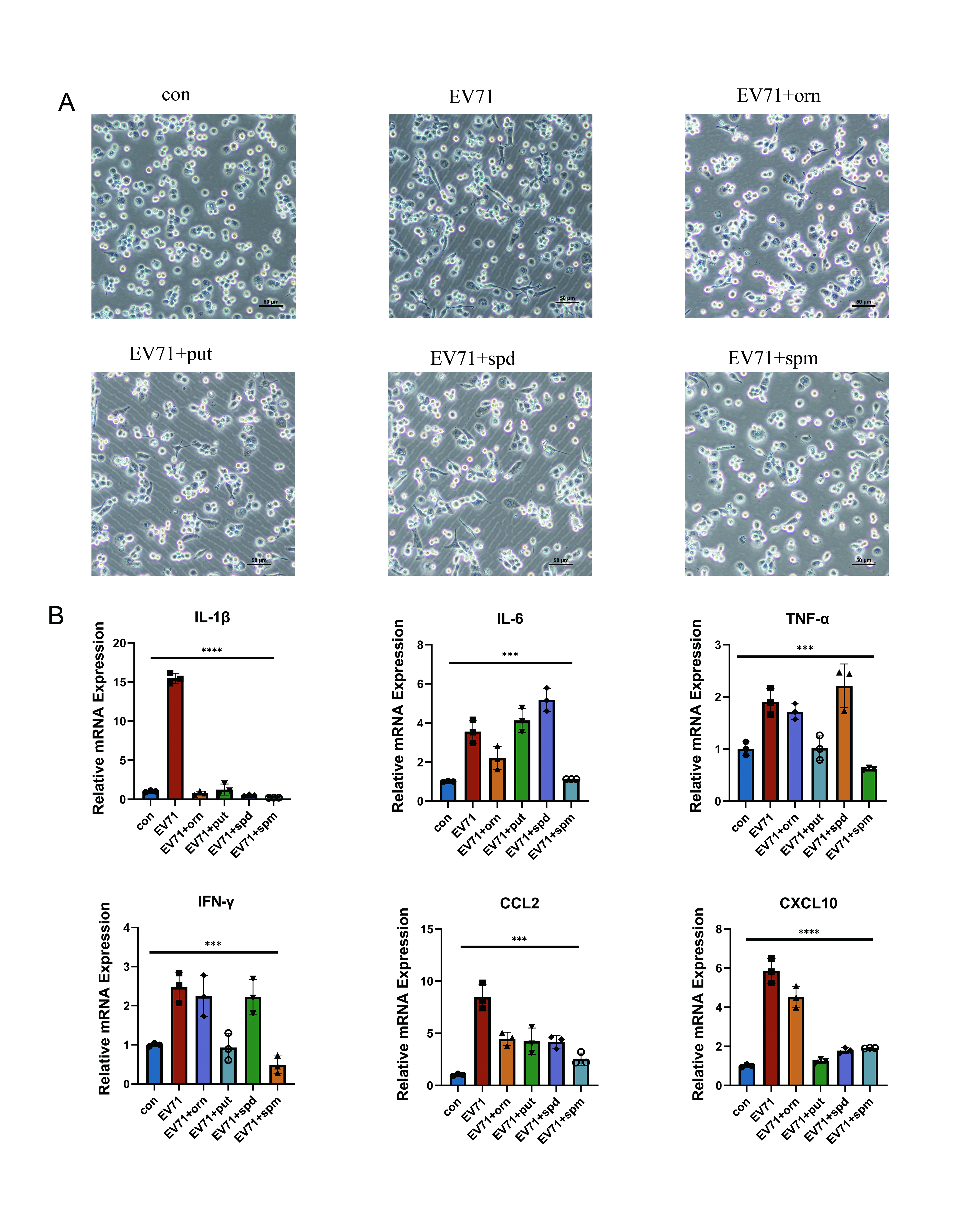

Supplement: Supplementary Figure 1 — Arginine metabolites inhibit cytokine storms in macrophages. (A)Arginine metabolites(ornithine:orn; putrescine:put; spermidine:spd; spermine:spm) affect the microscopic morphology of macrophages treated with EV71; (B)Arginine metabolites reduced the expression of EV71-induced IL-1β, IL-6, TNF-α, IFN-γ, CCL2 and CXCL10 in macrophages. (*p<0.05,**p<0.01,***p<0.001, ****p <0.0001). [file Image_1.tif]
